# Novel 3D volumetric and gallium casting assessment of anterior clinoidectomy for microsurgical clipping of posterior communicating artery aneurysms: an anatomical laboratory study

**DOI:** 10.1007/s00701-026-07011-1

**Published:** 2026-10-15

**Authors:** Diego Morales Roccuzzo, Lena Mary Houlihan, David Naughton, Shadi Bsat, Mohammadmahdi Sabahi, Ryan Gordon, David Monterroso-Cohen, Hamid Borghei-Razavi, Badih A. Adada

**Affiliations:** 1 Department of Neurological Surgery, Pauline Braathen Neurological Center, Cleveland Clinic Florida, 2950 Cleveland Clinic Blvd., Weston, FL 33331 USA; 2 Salford Royal Hospital, Manchester, UK

**Keywords:** Anterior clinoid process, Posterior communicating artery, Aneurysm, Clinoidectomy, Skull-base laboratory, Neurovascular anatomy, Microneurosurgical anatomy

## Abstract

**Background:**

Microsurgical treatment of dysmorphic, wide-neck posterior communicating artery aneurysms may require anterior clinoidectomy to improve exposure, proximal control, and clip maneuverability. Although many posterior communicating artery aneurysms are increasingly treated endovascularly, selected complex aneurysms continue to require microsurgical clipping. This anatomical laboratory study evaluates the effect of anterior clinoidectomy on the surgical corridor using 3D volumetric analysis and gallium casting.

**Materials and methods:**

Five formalin-fixed, color latex-injected cadaveric specimens were subjected to a pterional approach. Dissection and anterior clinoidectomy were performed microscopically with neuronavigation. For each dataset, 17 Cartesian coordinates were acquired to model the volume of surgical freedom (VSF) before and/or after clinoidectomy. The resulting 3D models were compared, and gallium casting was used as a complementary physical visualization of the postclinoidectomy surgical space.

**Results:**

Neuronavigation produced four paired pre-/postclinoidectomy datasets (left and right sides of two specimens; eight sets of 17 Cartesian coordinates) and six additional postclinoidectomy datasets, for a total of 14 coordinate sets. Mean VSF increased from 48.1 cm3 (48.1 mL) before clinoidectomy to 54.2 cm3 (54.2 mL) after clinoidectomy; this difference did not reach statistical significance (*P* = 0.304). Gallium casting provided a physical representation of the postclinoidectomy surgical field, which approximated an irregular two-based pyramid/frustum.

**Conclusion:**

In this cadaveric laboratory model, anterior clinoidectomy was associated with an increase in measured working volume and provided additional operative maneuverability for the microsurgical clipping corridor of posterior communicating artery aneurysms. These exploratory findings support the feasibility of 3D VSF modeling and gallium casting as complementary tools for skull-base laboratory training and future preoperative planning research.

**Supplementary Information:**

The online version contains supplementary material available at https://doi.org/10.1007/s00701-026-07011-1.

## Introduction

The anterior clinoid process (ACP) has traditionally served as a cornerstone for skull base surgeons operating in the middle and anterior base in the vicinity of the cavernous sinus and the sellar, parasellar, and suprasellar compartments. Removal of this structure (anterior clinoidectomy – “AC”) is a widespread procedure for aneurysm surgery, most of which arises from the Internal Carotid Artery (ICA) in the paraclinoidal area but is equally transposable to skull-based surgery and tumor resection for paracavernous pathology [35]. It plays a pivotal role in posterior communicating artery (PcoA) aneurysm surgeries, which might necessitate improved maneuverability [7, 17, 23].

The present study aims to determine how AC impacts the total volume of surgical freedom (VSF) during pterional craniotomy by quantifying changes in the surgical corridor with 3D volumetry and gallium casting [3, 9, 14, 15, 17, 18, 20, 22, 23, 26, 28–30, 32–34, 37, 42].

## Material and methods

### IRB/ethics committee and patient consent

For this study, IRB/ethics committee approval and patient consent for the procedure were not required or sought because they are not applicable since this work focused mainly on cadaveric investigations in the Skull Base Laboratory. The scarce clinical imaging utilized is completely anonymized and serves only for the purpose of in situ depiction of the pathology.

### Preliminary cadaveric preparation and positioning

This work involves analysis of the clinoidal region; five formalin-fixed, color latex-injected cadaveric specimens preserved in 70% ethanol solution were subjected to a pterional approach in a skull-base laboratory. Dissection and clinoidectomy were performed under a surgical microscope (Leica Microsystems Inc., IL, USA) using a high-speed drill (Stryker Instruments, MI, USA) with the aid of a Curve neuronavigation platform (Brainlab, Munich, Germany).

### Cartesian coordinates acquisition

**Fig. 1:**
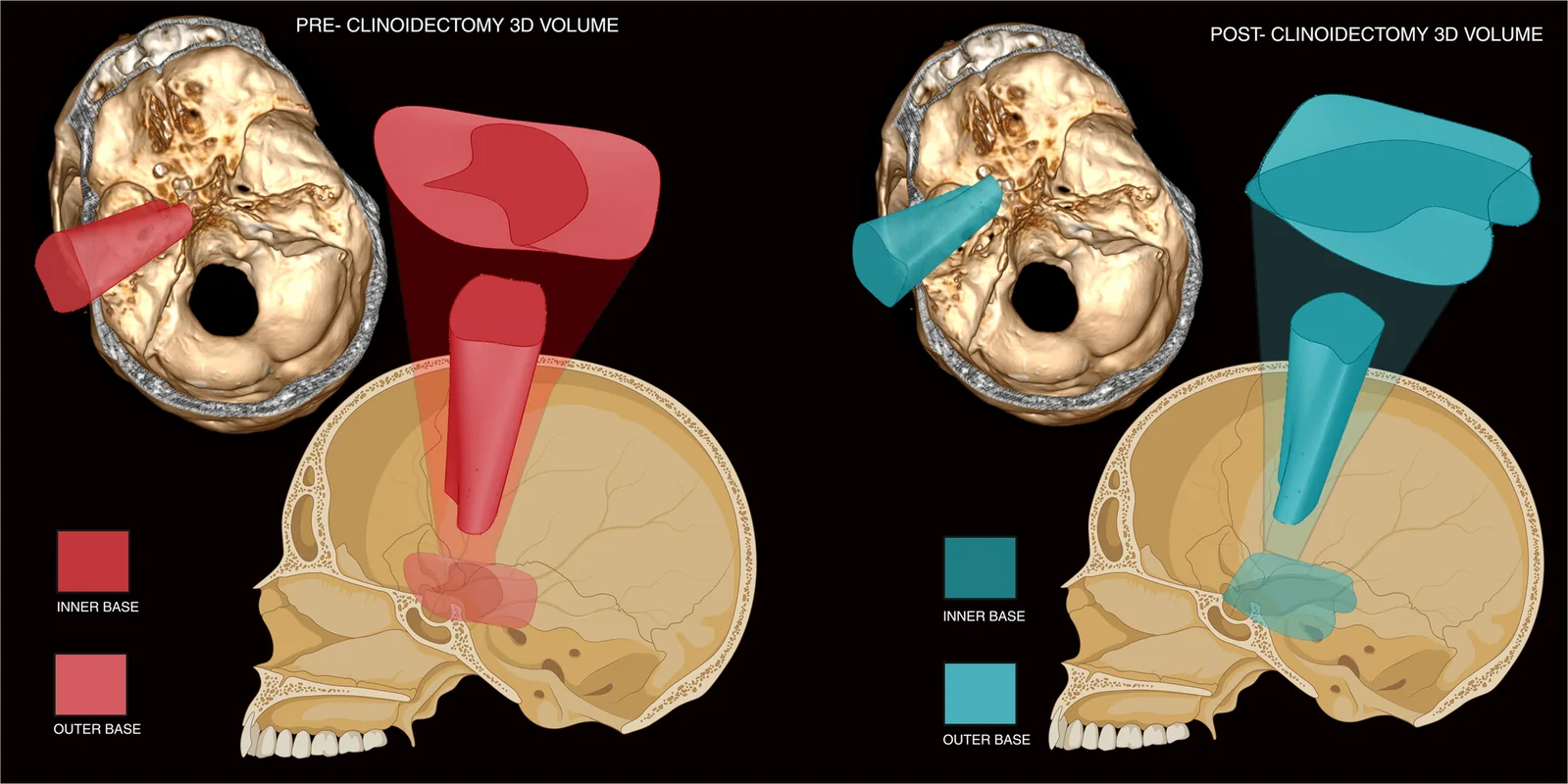
3D generated models of both corridors shown in a schematic manner

Coordinate-based acquisition of VSF was used to generate 3D models comparing pre- and postclinoidectomy volumes. By redefining the cornerstone method established by Houlihan et al., this work considers the surgical space as a two-based irregular frustum of a cone or an irregular two-based pyramid. Instead of acquiring 8 points (XYZ coordinates) to produce a base for the surgical space, we went one step further and navigated through 17 points: 1 point for the surgical target structure (STS), which will act as a center of our 3-dimensional figure (in our case, a point 4 mm posterior to the tip of the ACP); 8 points for the inner (medial) base surrounding the STS; and 8 points correlated to the outer (lateral) base [20] (Fig. 1).

### Mathematical analysis

The surgical corridor was modeled as the frustum of an irregular cone, with the surgical target structure on the narrower end of the frustum. To model the surgical corridor and measure a representative volume, 17 data points were measured for each dataset: 1 point for the surgical target structure (in our case, a point 4 mm posterior to the tip of the ACP), 8 points for the inner (medial) base surrounding the surgical target structure, and 8 points correlated to the outer (lateral) base [20].

To calculate the frustum, the central axis of the surgical corridor was obtained as the line joining the surgical target structure to the centroid of the points of the outer (lateral) base. The centroid of the 8-point outer base = (x_outer_, y_outer_, z_outer_) was calculated by averaging the x, y and z coordinates of the 8 points:

$${x}_{c}= \frac{{x}_{1}+ {x}_{2}+ {x}_{3}+\dots + {x}_{8}}{8}$$

$${y}_{c}= \frac{{y}_{1}+ {y}_{2}+ {y}_{3}+\dots + {y}_{8}}{8}$$

$${z}_{c}= \frac{{z}_{1}+ {z}_{2}+ {z}_{3}+\dots + {z}_{8}}{8}$$

The central axis vector $\overrightarrow{C}$ was calculated as the difference vector between the surgical target structure (x_sts_, y_sts_, z_sts_) and the centroid of the outer base (x_outer_, y_outer_, z_outer_):

$$\overrightarrow{c}=({x}_{c}, {y}_{c}, {z}_{c})=\left({x}_{sts}, {y}_{sts}, {z}_{sts}\right)- \left({x}_{outer}, {y}_{outer}, {z}_{outer}\right)$$

The two ends of the frustum were then modeled as planes perpendicular to this central axis, one containing the surgical target structure and one containing the centroid of the outer points. In the parametric equation of a plane (Ax + By + Cz + D = 0), the values of A, B and C are defined by the x, y and z coordinates of a vector perpendicular to the plane, in our case, the central axis vector of the surgical corridor $\overrightarrow{C}$. The values of D for the plane containing the surgical target structure (D_sts_) and the centroid of the outer points (D_c_) were calculated by rearranging the parametric equation.

$${D}_{sts}= -\left({x}_{c}{x}_{sts}+{y}_{c}{y}_{sts}+ {z}_{c}{z}_{sts}\right)$$

$${D}_{c}= -({x}_{c}{x}_{outer}+{y}_{c}{y}_{outer}+ {z}_{c}{z}_{outer})$$

Having calculated the parametric equations of the two planes, the 8 inner data points measured as the limits of maneuverability around the STS were translated onto the plane through the STS via the translation method described in Houlihan et al. [20]. The 8 outer data points measured as the limits of maneuverability were translated onto the plane through the centroid of the outer point via the same method. In both cases, the points were translated onto the plane along a line perpendicular to the plane and through the point.

The translation results in a frustum of an irregularly shaped cone, where the base and the top of the cone are parallel planes, and all 17 of the data points have been translated onto one of the two planes. These data were then used to create a 3D model of the corridor for each dataset in SolidWorks, and the volume of the structure was calculated by the software [21, 25, 38, 45].

### Gallium casting technique

**Fig. 2:**
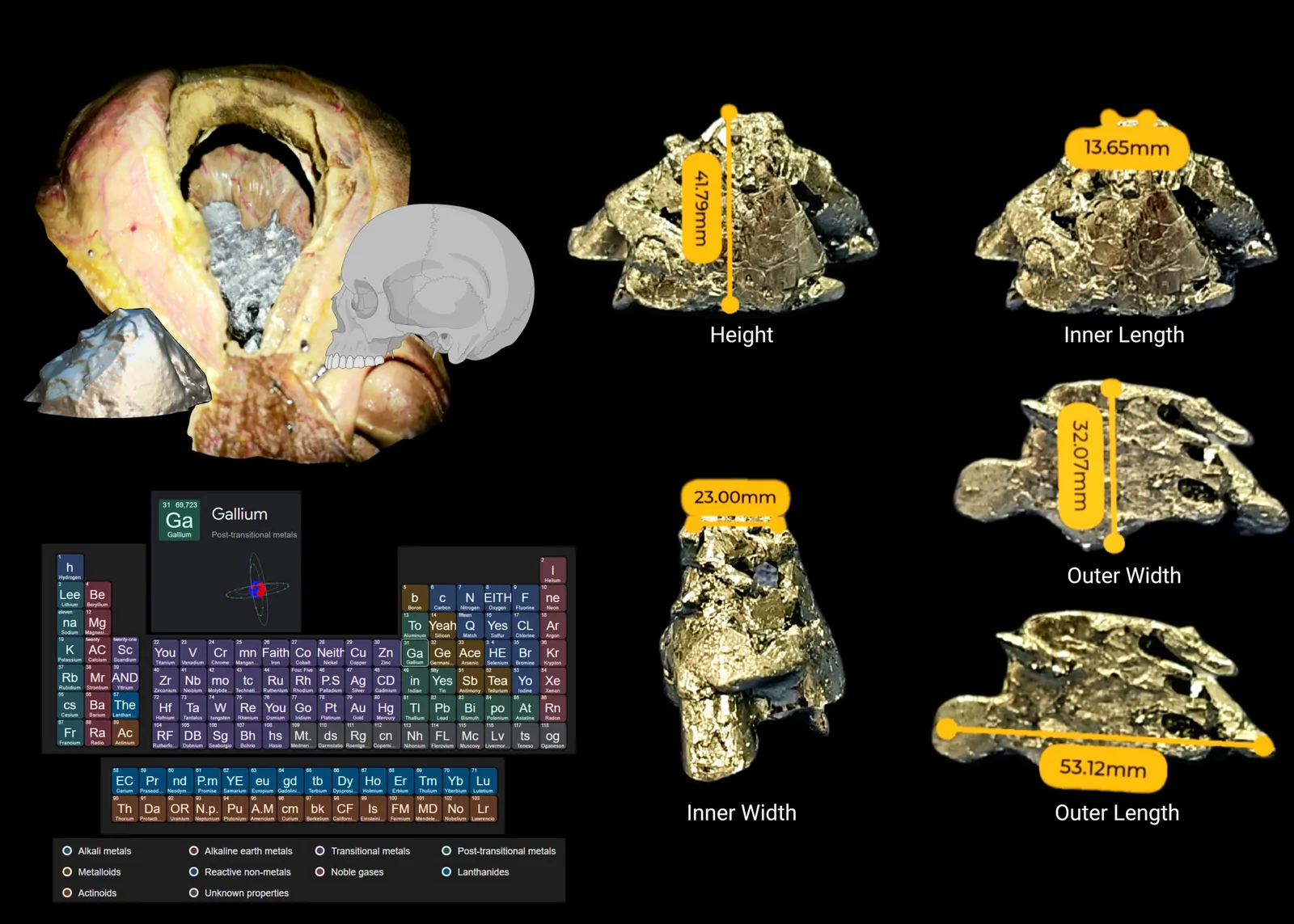
Solid gallium cast of the surgical space following clinoidectomy. The cast is larger than the 3D model because liquid-state gallium spreads across the extensive Sylvian fissure, resulting in an increased outer diameter. Link to an interactive virtual model acquired through photogrammetry of the cast: https://skfb.ly/oUrDr

This hypothesis was further explored using liquid gallium to create a cast of the surgical space. Gallium was selected for this laboratory technique because of its low melting point (approximately 30 degrees C), nonradioactive nature, and commercial availability. The gallium was melted in a vessel with boiling water and then poured into cellophane lining the surgical cavity after clinoidectomy. The specimen was then placed into a freezer at −13 degrees C for 25–30 min. After solidification, the gallium cast was extracted from the surgical cavity, subjected to photogrammetry and measurements (Polycam Inc., Minnesota, USA), and uploaded to a server to access an interactive 3D render of the gallium cast (Sketchfab Inc., Paris, France) (Fig. 2).

## Results

### Additional angle of attack

**Fig. 3:**
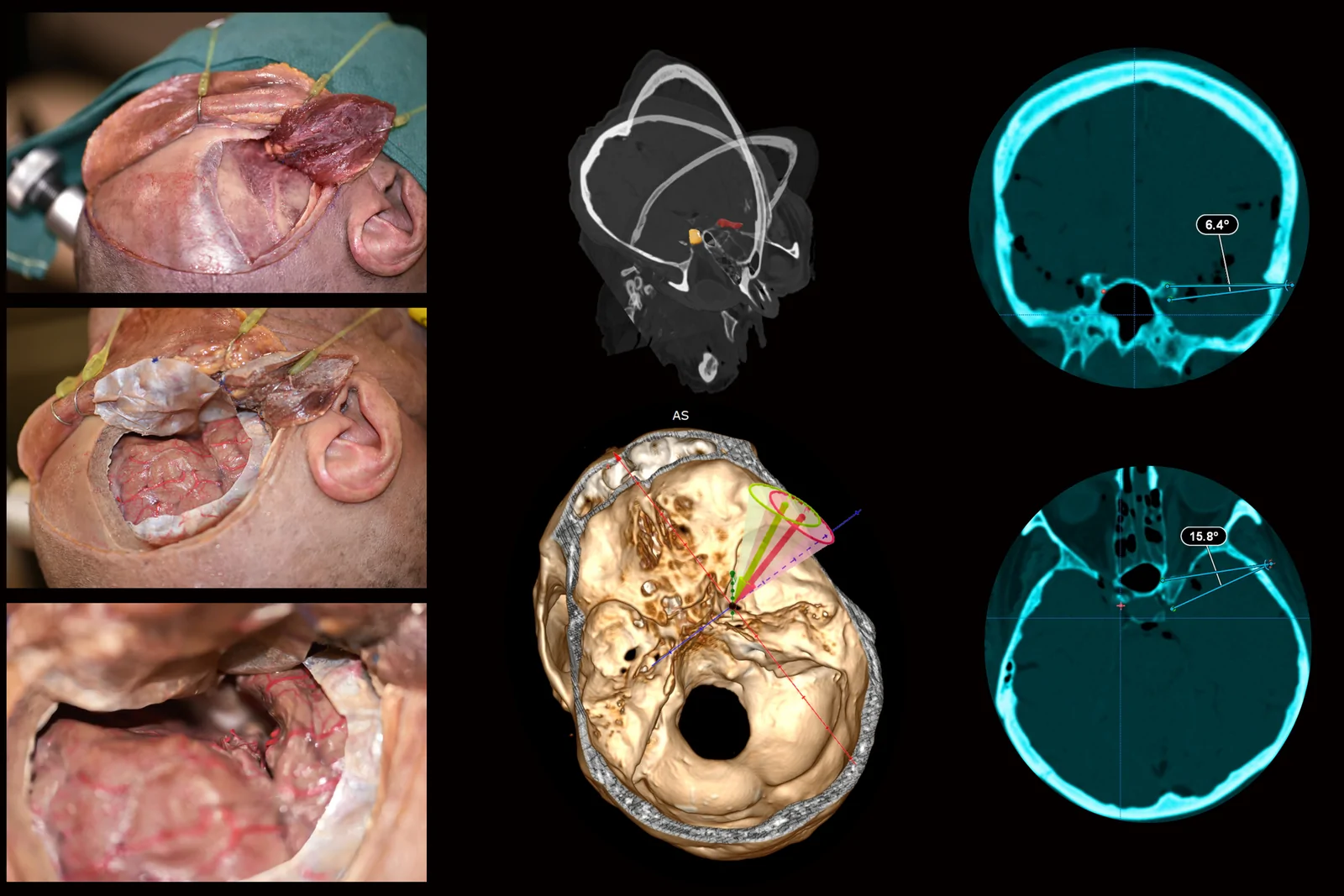
Stepwise anatomical dissection and navigation showing the hypothetical additional angle of attack gained when performing clinoidectomy

Through navigation of the anterior clinoid process in the specimens, a hypothetical additional angle of attack was acquired, namely 15.8 degrees mediolaterally and 6.4 degrees craniocaudally, taking into consideration the inner angles of attack one would obtain after clinoidectomy. Although the STS for this study was arbitrarily located 4 mm behind the tip of the ACP, this method helps explore the hypothesis of an increased theoretical angle and a different vector for the surgical corridor. The study did not calculate comprehensive angles of attack for the pterional approach because this was beyond its scope. Several studies have investigated angles of attack through different approaches and advanced morphometry, as discussed below (Fig. 3).

### Volume measurements

**Fig. 4:**
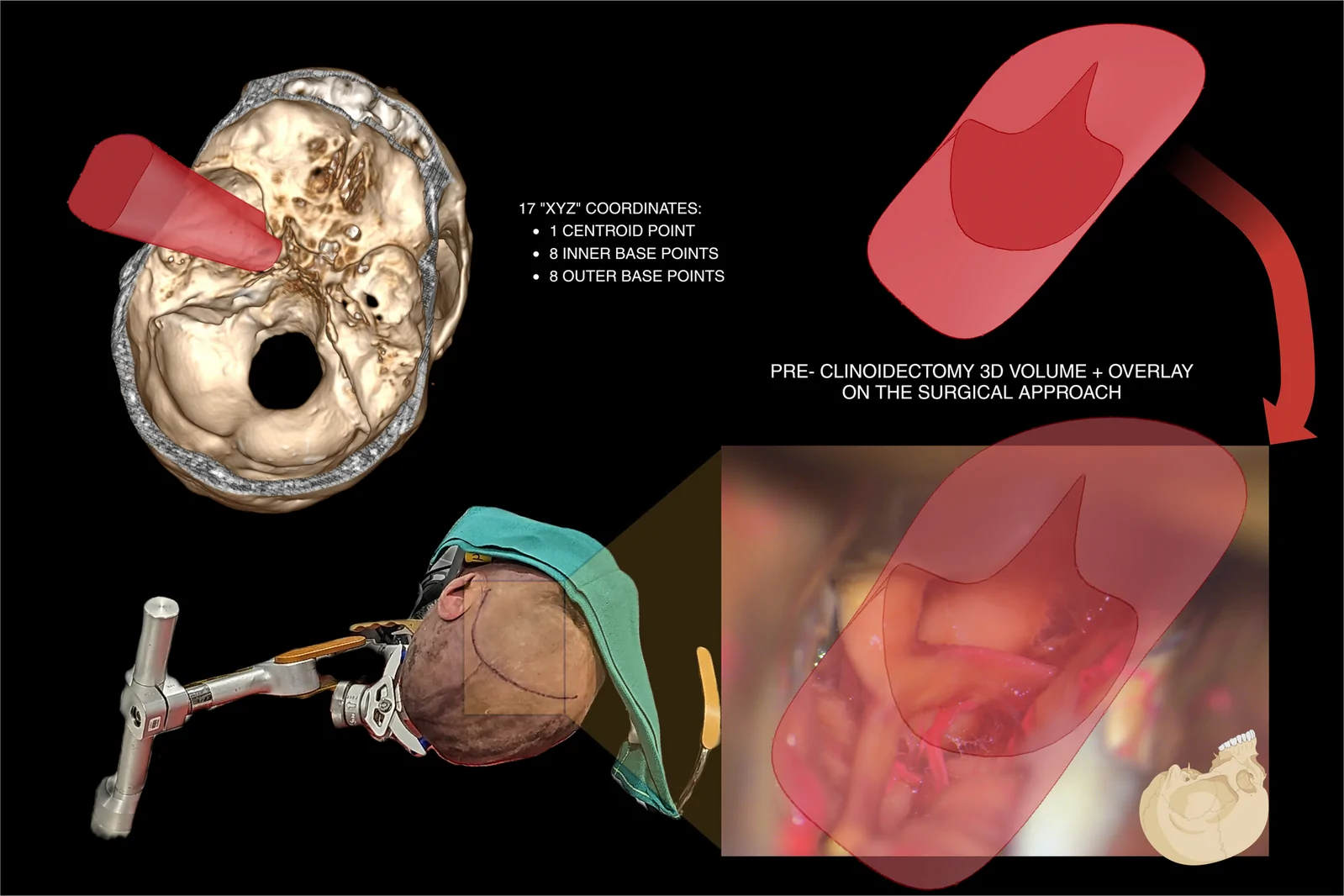
Surgical corridor of the preclinoidectomy scenario. Overlay of the model with the corresponding dissection obtained at the skull base laboratory

**Fig. 5:**
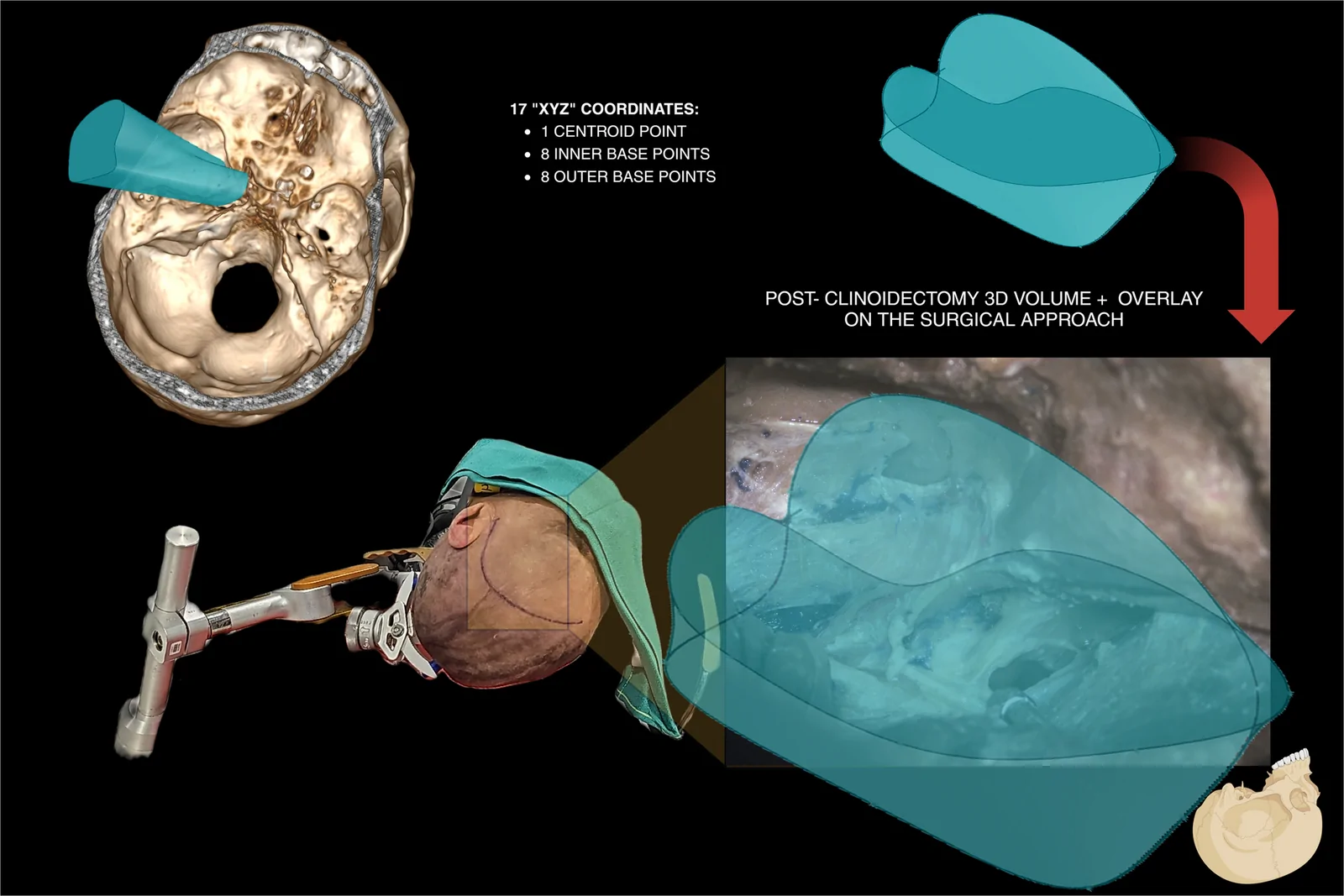
Surgical corridor of the postclinoidectomy scenario. Overlay of the model with the corresponding dissection obtained at the skull base laboratory

Neuronavigation produced four paired pre-/postclinoidectomy datasets from the left and right sides of specimens 1 and 2 (eight sets of 17 Cartesian coordinates) and six additional postclinoidectomy datasets, for a total of four preclinoidectomy and ten postclinoidectomy VSF measurements. Software processing rendered 3D shapes representing the surgical corridor for each scenario. The modified approach yielded a mean volume of 48.1 cm^3^ (48.1 mL; 48,053 mm^3^) for the preclinoidectomy scenario and 54.2 cm^3^ (54.2 mL; 54,240 mm^3^) for the postclinoidectomy scenario (*P*=0.304) (Table 1; Figs. 4 and 5).

**Table 1:** Sets of all the Cartesian coordinates for acquisition of every single rendered model (X, Y, Z)

Points	Specimen 1 left side		Specimen 1 right side		Specimen 2 left side		Specimen 2 right side
	Pre clin	Post clin	Pre clin	Post clin	Pre clin	Post clin	Pre clin
CENTER POINT	(16.5, −168.3, 685.1)	(16.5, −168.3, 685.1)	(−16, −167.7, 686)	(−16, −167.7, 686)	(17, −170, 689)	(17, −170, 689)	(−16, −171, 690)
POINT 1	(16.5, −174.1, 686)	(16.7, −184.2. 690.9)	(−16, −173.9, 687)	(−16.7, −187, 688.9)	(17.5, −171, 690)	(17.5, −186.7, 690)	(−16, −172.1, 690)
POINT 2	(19.2, −166.7, 682.1)	(19.2, −166.7, 682.1)	(−18, −166.1, 684)	(−19.2, −165, 683)	(18.2, −168, 684)	(18, −167, 684)	(−17, −169, 684)
POINT 3	(20.8, −175, 680.7)	(20.8, −175, 680.7)	(−21, −174.2, 681)	(−20.8, −175, 679)	(19, −175, 683)	(18, −174, 681)	(−18, −176, 683)
POINT 4	(18.7, −175.1, 692.9)	(19.6, −163, 694.3)	(−19, −174.4, 693)	(−19.6, −171, 693)	(19.3, −176, 696)	(18.7, −177, 695)	(−19, −177, 696)
POINT 5	(19.6, −163, 694.3)	(16.1, −156.8, 685)	(−19, −162, 695)	(−16.1, −153.4, 682)	(20, −165, 697.3)	(19, −164, 695.7)	(−19, −163, 697.3)
POINT 6	(16.1, −156.8, 685)	(17.4, −156.8, 691.5)	(−16, −155, 684)	(−17.4, −154.7, 689.4)	(17, −155.3, 685)	(17.5, −154, 687)	(−16, −153.9, 685)
POINT 7	(15.4, −160.6, 678.5)	(16.7,- 181.8, 683.6)	(−15, −159, 679)	(−16.7, −183.6, 681)	(16.8, −161.6, 680)	(17.5, −159, 679)	(−17.2, −160, 680)
POINT 8	(17, −156.8, 691.5)	(18.7, −175.1, 692.9)	(−16.5, −156.8, 690)	(−18.7, −173, 690.7)	(18, −158.3, 693)	(19, −156.2, 690.1)	(−17.2, −157.8, 693)
POINT 9	(124.6, −156.2, 702.3)	(124.6, −156.2, 702.3)	(−125.4, −155, 704)	(−124.6, −155, 704)	(126, −157.9, 705.4)	(126, −157.9, 700.7)	(−125.3, −156.7, 705.4)
POINT 10	(124.8, −189.3, 700.6)	(124.8, −189.3, 700.6)	(−126, −190, 702)	(−124.8, −191, 701)	(126.3, −191, 702.7)	(126.3, −191, 699.8)	(−124.9, −189, 702.7)
POINT 11	(123.2, −163.2, 691.2)	(123.2, −163.2, 691.2)	(−122, −161.5, 693)	(−123.2, −164, 692.3)	(126, −165, 694)	(126, −165, 695)	(−125, −163, 694)
POINT 12	(121.3, −173.5, 681.2)	(121.3, −173.5, 681.2)	(−122.5, −171.3, 682)	(−121.3, −173, 681)	(123, −174.8, 683)	(123, −174.8, 680)	(−124, −175, 683)
POINT 13	(120, −175.6, 714.4)	(120, −175.6, 714.4)	(−121, −174.7, 716)	(−120, −173.5, 715.6)	(122, −177.1, 716)	(122, −177.1, 714.3)	(−124, −179, 716)
POINT 14	(125.4, −165.6, 710)	(125.4, −165.6, 710)	(−125, −163, 711.9)	(−125.4, −175, 711)	(126.2, −166.9, 713.7)	(126.2, −166.9, 710.2)	(−126.9, −164, 713.7)
POINT 15	(124.3, −166, 688)	(124.3, −166, 688)	(−125, −169, 689)	(−124.3, −164, 688.7)	(121.8, −168.3, 690)	(121.8, −168.3, 687.5)	(−123, −166.5, 690)
POINT 16	(126.5, −182.9, 708.4)	(126.5, −182.9, 708.4)	(−127, −184, 710)	(−126.5, −184, 711.5)	(125, −184, 710)	(125, −184, 709.5)	(−124, −183, 710)
VOLUMES	43,324,00	51,194,00	45,670,00	41,665,00	49,766,00	59,357,00	53,453,00

Points	Specimen 2 right side	Specimen 3 post clinoidectomy		Specimen 4 post clinoidectomy		Specimen 5 post clinoidectomy	
	Post clin	Left side	Right side	Left side	Right side	Left side	Right side
CENTER POINT	(−16, −171, 690)	(17, −169.2, 683)	(−15, −170.2, 687)	(15, −167.2, 685)	(−16.2, −169, 684)	(14.8, −169.2, 683)	(15.7, −170.1, 686)
POINT 1	(−16, −187.3, 690)	(17, −184.9, 685)	(−15, −182.7, 687)	(15, −187, 683)	(−16.2, −187, 684)	(14.8, −188, 686)	(18, −187.9, 687)
POINT 2	(−16.5, −167, 683)	(19.9, −168.2, 680)	(−16, −165.1, 680)	(17, −168, 679)	(−17.9, −165, 681)	(20, −167, 684)	(20, −168, 684)
POINT 3	(−17.8, −175, 682.1)	(21, −174, 679.2)	(−17, −173.5, 680.5)	(18.5, −175.7, 677.8)	(−17.3, −173.5, 675.9)	(20, −175, 680.3)	(22, −175.7, 682.3)
POINT 4	(−18.7, −176.4, 695)	(19.1, −173.1, 690)	(−17, −173.1, 690)	(17, −164, 693.2)	(−16.4, −163, 691.5)	(19, −160, 693.5)	(20, −162, 693.5)
POINT 5	(−19, −162, 695.7)	(20, −161.4, 692.2)	(−18.9, −160, 690.5)	(18, −157, 690.5)	(−16.5, −151.9, 689)	(19, −153.5, 691)	(21, −152.1, 694)
POINT 6	(−16, −152, 687)	(16.7, −150.3, 684.7)	(−18.9, −152.7, 686)	(16, −159, 682.8)	(−16.7, −157, 680.5)	(18, –156, 685)	(19.5, −157.8, 686)
POINT 7	(−17, −158, 678)	(16, −159, 675.6)	(−15, −157, 678)	(15,- 185.6, 672)	(−14.9, −183.7, 675)	(17,- 184.1, 679.2)	(17,- 185, 677.5)
POINT 8	(−18, −155.5, 691)	(17.8, −153, 689.8)	(−16, −151.5, 693)	(15.9, −178.4, 688)	(−16.7, −180, 690)	(16.5, −175, 688.2)	(17.4, −175.4, 692)
POINT 9	(−125.3, −155.6, 707)	(122.9, −155.7, 702.3)	(−121, −157, 703.5)	(125, −162, 700)	(−123, −161, 701)	(124, −155, 705.1)	(121, −156, 700.5)
POINT 10	(−125, −191.8, 705.3)	(122, −187, 700.6)	(−120.6, −185.4, 702)	(124, −185, 699)	(−123.5, −184, 700)	(123, −189, 704)	(121.5, −187, 701)
POINT 11	(−123, −161.5, 692)	(121.9, −160.8, 691.2)	(−120.7, −163, 690.5)	(124, −165, 690)	(−124.2, −168, 695)	(122, −161, 693.5)	(122, −164, 694)
POINT 12	(−123, −174, 685)	(120.2, −171.6, 681.2)	(−121, −173, 683.7)	(123, −172.2, 679.1)	(−124, −174.5, 682)	(124, −172.9, 683.5)	(121.5, −170, 683)
POINT 13	(−125, −181, 717.5)	(119, −174.2, 714.4)	(−120, −176, 716.8)	(123, −172, 710)	(−121.6, −174.2, 708)	(120, −175.3, 717.9)	(120, −173, 712)
POINT 14	(−127.5, −165.2, 711.3)	(123.5, −163.9, 710)	(−124, −165, 712)	(125, −165, 712)	(−125, −163.8, 709)	(125, −165.9, 712)	(124 −167, 708)
POINT 15	(−122.2, −166, 691)	(122.7, −165, 688)	(−121.5, −164, 690)	(126, −163.6, 690)	(−124, −162.6, 689)	(124.8, −167, 689)	(121, −164, 686.5)
POINT 16	(−122, −185, 710)	(123.9, −180.3, 708.4)	(−122.6, −179.5, 710.5)	(124, −181, 710)	(−123.3, −180, 713)	(124.5, −183, 709.7)	(124 −183.3, 705.9)
VOLUMES	64,743,00	56,262,00	49,413,00	53,649,00	43,701,00	55,059,00	47,809,00

Volume of each rendered model (mm^3^ = Cubic millimeters)

### Exploratory statistical comparison of VSF

In the first analysis, preclinoidectomy and postclinoidectomy volumes were compared with paired t tests using the four paired datasets available from the left and right sides of specimens 1 and 2.

In the second analysis, preclinoidectomy and postclinoidectomy measurements were treated as two distinct groups by comparing the mean of the four preclinoidectomy volumes with the mean of all ten postclinoidectomy volumes. This comparison did not demonstrate a statistically significant difference between groups (*P* = 0.293). Because distributional assumptions could not be verified for the unpaired comparison, the Mann–Whitney U test was performed as a nonparametric sensitivity analysis. Although the mean VSF was higher after clinoidectomy, this difference did not reach statistical significance (*P* = 0.304).

These results are reported as exploratory and should not be interpreted as confirmatory statistical evidence of a volumetric effect.

### Gallium casting as a novel feasible technique in skull-base research

Gallium casting provided a complementary physical visualization of the postclinoidectomy surgical field, which was shaped as an irregular two-based pyramid or frustum of a cone. The solid gallium cast of the surgical space following clinoidectomy was larger than the corresponding 3D model because liquid-state gallium spread across the extensive Sylvian fissure, increasing its outer diameter.

(Video 1).

## Discussion

### Geometry and topology of neurosurgical approaches

Surgical approaches to the anterior clinoidal region are defined by specific features, including a superficial area for instrument entry, a deep area at the surgical target, height (the distance between superficial and deep areas), volume, and trajectory. The concept of surgical freedom uses VSF as a metric for evaluating surgical access. Specific studies have compared volumetric measurements of pterional craniotomies with different cranial approaches, even providing accurate mediolateral and craniocaudal angles of attack [20]. Many endeavors in this area have shed light on this matter, especially summarizing and gathering all the relevant data and concepts that lay the groundwork for several studies, such as the comprehensive study carried out by Doglietto et al. [1, 13]. First, Honeybul et al. pioneered this area by directly measuring the surgical window and placing wires at the level of the craniotomy [19]. Similarly, Alaywan and Sindou introduced the concept of the “field-of-view angle” [2]. Schwartz et al. used a frameless stereotactic device to register XYZ coordinates, thus developing the “cone-of-view” primordial concept [41].

In general, a neurosurgical approach can be described as a truncated pyramid [1, 13]. Houlihan et al. further developed VSF as a critical metric for surgeons, offering a spatially precise 3D model of the surgical corridor. In their study, a novel methodology was developed to provide surgeons with a comprehensive and precise metric for assessing access corridors. This approach models the surgical corridor as a cone with an irregular base acquired from 8 neuronavigated points in different planes and then calculates the volume of this cone, thereby quantifying surgical freedom in a 3-dimensional manner. A normalized VSF corrects for measurement inaccuracies and provides a consistent reference metric for cross-study comparisons. It overcomes several limitations in existing quantitative assessment methods, enabling the creation of 3-dimensional models of surgical corridors and setting a higher standard for evaluating surgical freedom. Comparative results of measurements for specific anatomical structures and surgical approaches have indicated that pterional craniotomy generally offers a larger VSF than does, for instance, supraorbital craniotomy for accessing various structures. The surgical view of the cadaveric anatomy, which is consistent with the surgical corridor model parameters, with the use of the paraclinoidal ICA as an STS revealed a normalized posterior approach VSF of 165.88 mm^3^, with a craniocaudal angle of attack = 36.72 and mediolateral angle of attack = 50.55. This information can assist surgeons in selecting the most suitable approach on the basis of their specific needs. The researchers went even further and selected three hypothetical surgical target structures for simulating different real-life scenarios: the paraclinoid ICA, the terminal ICA, and the anterior communicating artery. [8, 10, 12, 15, 20, 43] [21, 25, 38, 45]

Prior morphometric studies provide complementary context: Evans et al. quantified changes in the optic nerve, internal carotid artery, and opticocarotid triangle before and after AC, whereas Beretta et al. measured exposure of the sellar and perisellar regions across minimally invasive and standard approaches [8, 15, 16, 39]. Although related to other approaches, work from Alvernia et al. also provided insight and lateral thinking into the traditional way we approach surgical pathways. [4–6, 11, 24, 27, 36, 40, 44].

### Predicting the need for clinoidectomy

**Fig. 6:**
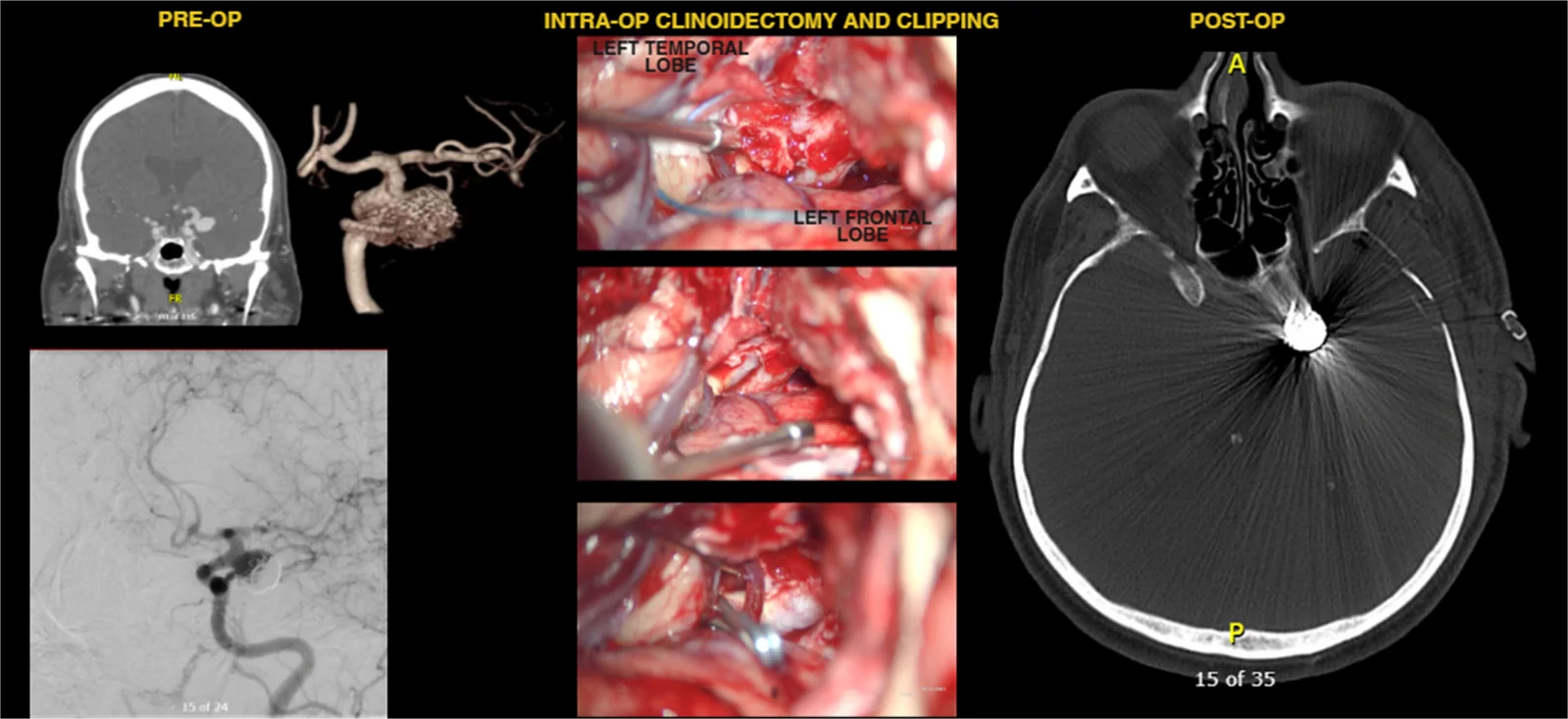
Preoperative CT angiography and DSA demonstrate a highly complex left PcoA aneurysm with partial endovascular embolization. Intraoperative imaging documents the AC during the left cranio-orbitozygomatic approach: the dura propria of the temporal lobe is peeled off the lateral wall of the cavernous sinus, allowing visualization of the anterior clinoid process. The optic canal is subsequently unroofed, followed by drilling of the optic strut and complete removal of the anterior clinoid process, thereby expanding the surgical corridor and maximizing exposure of the supraclinoidal ICA. Postoperative brain CT confirms successful microsurgical clipping of the aneurysm at the supraclinoidal ICA, with the absence of the anterior clinoid process clearly visible

**Fig. 7:**
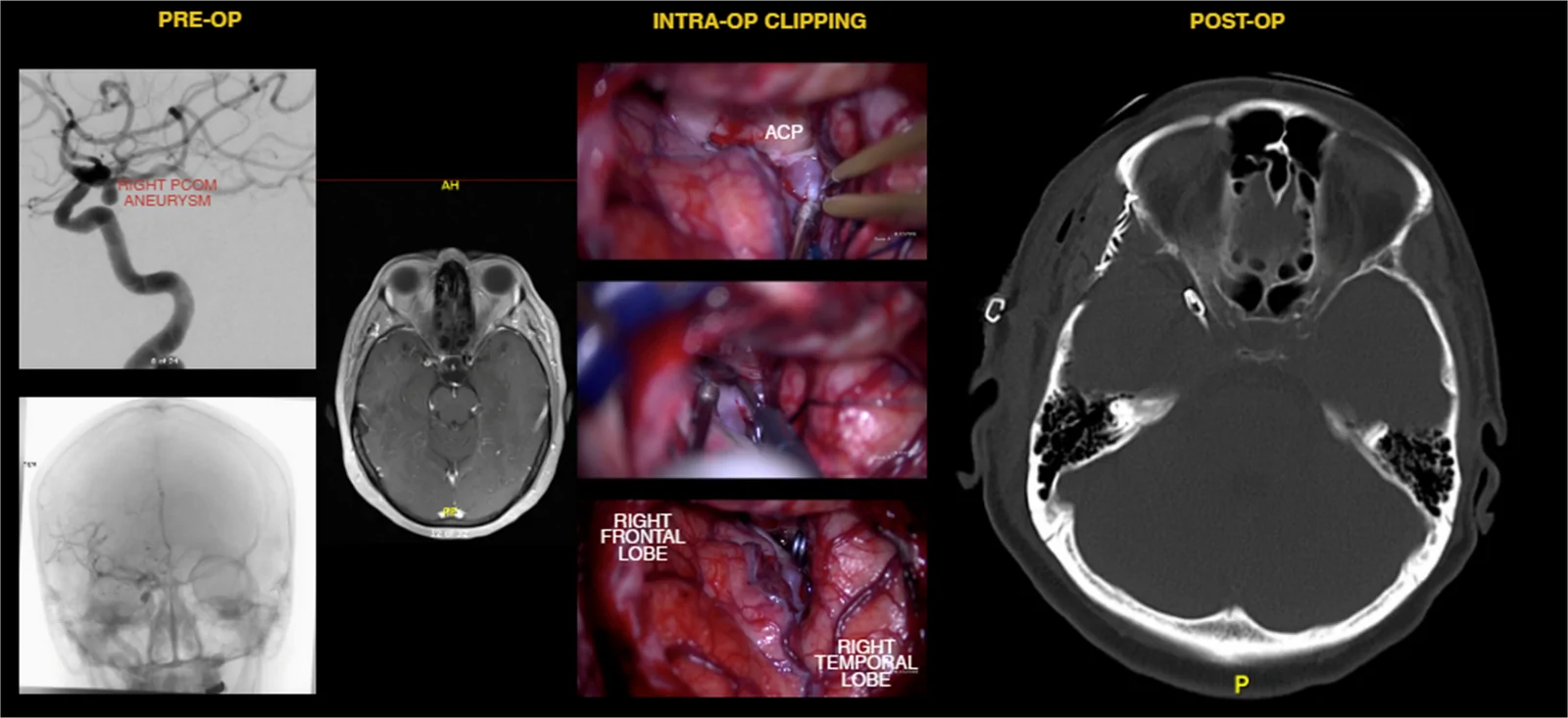
Right pterional craniotomy for microsurgical clipping of a PcoA aneurysm. Preoperative digital subtraction angiography and angio-MRI confirm the aneurysm. Following a standard pterional craniotomy and drilling of the lesser sphenoid wing, the Sylvian fissure was opened in an inside-to-outside fashion to expose the carotid bifurcation and optic nerve. Microsurgical dissection facilitated identification of the anterior choroidal artery and the oculomotor nerve, situated inferior to the aneurysm dome. After mobilizing the aneurysm from the mesial temporal lobe, a 7-mm straight permanent clip was applied across the neck, followed by aneurysm decompression. Postoperative CT imaging confirms successful exclusion of the aneurysm with the clip positioned at the supraclinoidal carotid artery and the anterior clinoid process remaining intact

Among the anatomical factors identified in the reviewed literature that may help predict the need for AC, the most practical is the distance between the tip of the anterior clinoid process and the proximal neck of the aneurysm on CTA. Prior work reported that this distance was shorter in the clinoidectomy group than in the non-clinoidectomy group (4.4 ± 0.7 mm vs. 7.2 ± 1.4 mm [7, 9, 15, 17, 31, 35]), suggesting that AC is rarely needed when the distance exceeds 5.8 mm and is more frequently required at shorter distances [35]. Although other morphometric parameters have been described, they are less practical for routine decision-making because they can be difficult to obtain reliably on digital subtraction angiography. The data also indicate that the communicating segment of the ICA takes a more lateral course in the clinoidectomy group. The orientation of an aneurysm dome in relation to the parent artery remains important for surgical planning because different orientations may require specific surgical strategies and may be associated with distinct complications. To illustrate the practical application of our anatomical findings, we incorporated two representative clinical cases involving microsurgical repair of complex left and right posterior communicating artery (PcoA) aneurysms via left cranio-orbitozygomatic and right pterional craniotomies, respectively. These cases, one requiring AC and one in which the clinoid was preserved, provide clinical correlation to the volumetric data. By presenting preoperative imaging, intraoperative documentation of clinoidectomy, and postoperative imaging showing the clip position relative to the supraclinoidal carotid artery, we aim to demonstrate how laboratory-rendered models of surgical freedom may translate to complex vascular challenges in the operating room (Figs. 6 and 7; Video 2).

### Surgical anatomy and nuances of the pcoa and anterior clinoidectomy

The anatomy of the PcoA complex can vary significantly [23, 33]. The size of the PcoA artery can range from small and nearly invisible on angiography to large and similar in size to the posterior cerebral artery (PCA). Fetal PcoA variants, with a diameter similar to the P2 segment of the PCA and a smaller or atrophic P1 segment, are important to consider during procedures such as aneurysm clipping or coiling. True PcoA Aneurysms: True PcoA aneurysms are rare but require special attention. They originate solely from the PcoA artery and represent a small percentage of all aneurysms. Preserving blood flow in the PcoA artery during treatment is critical.

AC may be necessary for selected PcoA aneurysms when proximal control, neck exposure, or clip application is limited by the ACP and adjacent dural rings. Given the technical intricacy of the maneuver and its proximity to critical structures, careful preoperative assessment is essential. Removal of the ACP can improve exposure around the optic nerve and intracranial ICA while reducing the need for brain retraction and increasing the mobility of adjacent neurovascular structures. Although endovascular treatment is frequently preferred for many PcoA aneurysms, microsurgical clipping remains important for selected complex lesions. The decision to perform AC should therefore be individualized based on aneurysm morphology, relationship to the ACP, available proximal control, and the anticipated clipping corridor.

Successful AC requires careful evaluation of several anatomical variations and landmarks. Preoperative imaging should assess ossification of the carotico-clinoid and interclinoid ligaments, while intraoperative orientation relies on identification of key structures, including the meningo-orbital band (orbitotemporal periosteal fold), falciform ligament, petrosphenoidal and petrolingual ligaments, and the anterior and posterior petroclinoid ligaments, the latter closely related to the trochlear nerve.

Within this anatomical framework, our 3D volumetric analysis and Gallium casts provide quantitative evidence of the additional operative space created by AC. The increase in surgical freedom can be explained by removal of the ACP, which exposes the clinoid space and improves access to the clinoidal internal carotid artery, carotid cave, and adjacent dural rings while reducing the osseous constraints imposed by the optic strut. Furthermore, the Gallium casts illustrate the spatial relationship of the expanded corridor to critical neurovascular structures, including the carotid collar and carotico-oculomotor membrane. Together, these findings complement previous qualitative anatomical descriptions by providing objective volumetric measurements of the surgical exposure achieved through AC [3, 9, 14, 15, 17, 18, 22, 23, 26, 28–30, 32–34, 42].

## Strengths and limitations

This study establishes a sophisticated anatomical and methodological framework for surgical planning and microsurgical training. A primary strength of this research lies in the high-fidelity integration of 3D volumetric analysis with gallium casting techniques, providing detailed spatial insights into the surgical corridor associated with AC and posterior communicating artery aneurysm clipping. This combined approach offers a level of architectural detail that is difficult to achieve with conventional methods and serves as a robust reference for future laboratory and translational research.

Several limitations must be acknowledged. The use of formalin-fixed, latex-injected cadaveric specimens—while a standard in anatomical research—does not fully replicate the dynamic and physiological properties of living tissue. Fixation and injection processes may inevitably alter tissue characteristics and introduce artifacts, while the absence of blood flow and pulsatility limits the direct extrapolation of these data to in vivo surgical conditions.

The small number of specimens represents a primary limitation which restricts the statistical power of the analysis and precludes robust statistical generalization. However, it is essential to emphasize that the objective of this work is not population-level inference, but rather a meticulous anatomical and methodological exploration within a basic laboratory research framework. Within this scope, the qualitative and spatial insights gained from 3D volumetry and gallium casting provide a foundational understanding of microsurgical anatomy and operative corridors that remains highly valuable despite the limited sample size.

From a methodological perspective, the analysis focused specifically on the additional exposure and VSF afforded by AC rather than the full spectrum of surgical attack angles. While this targeted approach allowed evaluation of the clinoidectomy-specific contribution, it does not encompass the entirety of the surgical trajectory.

Finally, although many posterior communicating artery aneurysms are currently managed via endovascular techniques, detailed understanding of the microneurosurgical anatomy of this region remains vital for selected complex cases requiring surgical clipping. Future studies with larger specimen series, standardized acquisition protocols, and complementary clinical correlation are warranted to validate and expand upon these exploratory findings.

## Conclusions

The 3D virtual models rendered from neuronavigated coordinates demonstrated that the surgical corridor after AC can be represented as an irregular two-based cone, or conical frustum. In this cadaveric series, AC was associated with increased measured working volume and additional maneuverability through mobilization of key neurovascular landmarks involved in microsurgical clipping of posterior communicating artery aneurysms. Because the statistical analysis was exploratory and not confirmatory, these findings should be interpreted as methodological and anatomical observations rather than definitive proof of a volumetric benefit. The combined use of 3D VSF modeling and gallium casting may provide a useful platform for future skull-base laboratory training and preoperative planning research.

## Supplementary Information

Below is the link to the electronic supplementary material.

ESM 1 Supplementary Material 1: 3D model of gallium casting across the Sylvian fissure, acquired through photogrammetry. (MP4 8.37 MB)

ESM 2 Supplementary Material 2: Hands-on demonstration of the rendered models and the main hypothesis in the context of aneurysm surgery requiring AC, highlighting the inner (medial) base of the truncated cone that may contribute to surgical freedom in the deep surgical field. (MP4 46.4 MB)

ESM 3 Supplementary Material 3 (XLSX 14.6 KB)

## Data Availability

The authors confirm that the data supporting this study's findings are available within the article.
